# Incidence and outcome of invasive candidiasis in intensive care units (ICUs) in Europe: results of the EUCANDICU project

**DOI:** 10.1186/s13054-019-2497-3

**Published:** 2019-06-14

**Authors:** Matteo Bassetti, Daniele R. Giacobbe, Antonio Vena, Cecilia Trucchi, Filippo Ansaldi, Massimo Antonelli, Vaclava Adamkova, Cristiano Alicino, Maria-Panagiota Almyroudi, Enora Atchade, Anna M. Azzini, Novella Carannante, Alessia Carnelutti, Silvia Corcione, Andrea Cortegiani, George Dimopoulos, Simon Dubler, José L. García-Garmendia, Massimo Girardis, Oliver A. Cornely, Stefano Ianniruberto, Bart Jan Kullberg, Katrien Lagrou, Clement Le Bihan, Roberto Luzzati, Manu L. N. G. Malbrain, Maria Merelli, Ana J. Marques, Ignacio Martin-Loeches, Alessio Mesini, José-Artur Paiva, Maddalena Peghin, Santi Maurizio Raineri, Riina Rautemaa-Richardson, Jeroen Schouten, Pierluigi Brugnaro, Herbert Spapen, Polychronis Tasioudis, Jean-François Timsit, Valentino Tisa, Mario Tumbarello, Charlotte H. S. B. van den Berg, Benoit Veber, Mario Venditti, Guillaume Voiriot, Joost Wauters, Philippe Montravers

**Affiliations:** 1 Infectious Diseases Clinic, Department of Medicine University of Udine and Santa Maria Misericordia Hospital, Piazzale Santa Maria della Misericordia 15, 33100 Udine, Italy; 2 Department of Health Sciences, University of Genoa, Genoa, Italy; 3 Health Planning unit, Azienda Ligure Sanitaria della Regione Liguria (A.Li.Sa.), Liguria Region, Italy; 4 Health Planning unit, Policlinic San Martino Hospital - IRCCS, Genoa, Italy; 5 Department of Intensive Care and Anesthesiology, Università Cattolica del Sacro Cuore, Fondazione Policlinico Universitario Agostino Gemelli, Rome, Italy; 6 Clinical Microbiology and ATB Centre, Institute of Medical Biochemistry and Laboratory Diagnostics, General University Hospital, Prague, Czech Republic; 7 Department of Medical Microbiology, Medical Faculty of Palackeho University, Olomouc, Czech Republic; 8 Medical Direction, Santa Corona Hospital, ASL 2 Regional Health System of Liguria, Pietra Ligure, Italy; 9 Department of Critical Care, University Hospital Attikon, Medical School, University of Athens, Athens, Greece; 10 Département d’Anesthésie-Réanimation, CHU Bichat-Claude Bernard, HUPNVS, APHP, Paris, France; 11 Department of Diagnostics and Public Health, Infectious Disease Unit, University of Verona, Verona, Italy; 12 First Division, Cotugno Hospital, AO dei Colli, Naples, Italy; 13 Department of Medical Sciences, Infectious Diseases, University of Turin, Turin, Italy; 14 Department of Surgical, Oncological and Oral Science (Di.Chir.On.S.), Section of Anesthesia, Analgesia, Intensive Care and Emergency, Policlinico Paolo Giaccone, University of Palermo, Palermo, Italy; 15 Department of Anesthesiology and Intensive Care Medicine, Heidelberg University Hospital, Heidelberg, Germany; 16 Servicio de Cuidados Críticos y Urgencias, Hospital San Juan de Dios del Aljarafe, Bormujos, Sevilla Spain; 17 Department of Anesthesia and Intensive Care, University Hospital of Modena, Modena, Italy; 18 Department I of Internal Medicine, University Hospital of Cologne and Cologne Excellence Cluster on Cellular Stress Responses in Aging-Associated Diseases (CECAD), Cologne, Germany; 19 Infectious Diseases Unit, Department of Medical and Surgical Science, S. Orsola-Malpighi Hospital, University of Bologna, Bologna, Italy; 20 Radboud Center for Infectious Diseases, Radboud University Medical Center, Nijmegen, The Netherlands; 21 Department of Laboratory Medicine and National Reference Centre for Mycosis, University Hospitals of Leuven, Leuven, Belgium; 22 Department of Microbiology and Immunology, KU Leuven, Leuven, Belgium; 23 Bichat-Réanimation médicale et des maladies infectieuses, Medical ICU, Paris, France; 24 Infectious Diseases Department, Azienda Sanitaria Universitaria Integrata di Trieste, Trieste, Italy; 25 Department of Intensive Care Medicine, University Hospital Brussels (UZB), Jette, Belgium and Faculty of Medicine and Pharmacy, Vrije Universiteit Brussel (VUB), Brussels, Belgium; 26 C.H. Vila Nova de Gaia/Espinho, Vila Nova de Gaia, Portugal; 27 Department of Intensive Care Medicine, Multidisciplinary Intensive Care Research Organization (MICRO), St. James’s Hospital, Dublin, Ireland; 28 Hospital Clinic, IDIBAPS,CIBERes, universidad de Barcelona, Barcelona, Spain; 29 Department of Emergency and Intensive Care Medicine, Centro Hospitalar Universitário São João, Faculdade de Medicina da Universidade do Porto e Grupo de Infecção e Sépsis, Porto, Portugal; 30 Department of Biopathology and Medical Biotechnologies, Section of Anesthesia, Analgesia, Intensive Care and Emergency, Policlinico P. Giaccone, University of Palermo, Palermo, Italy; 31 Department of Infectious Diseases, Manchester University NHS Foundation Trust, Wythenshawe Hospital; and Faculty of Biology, Medicine and Health, University of Manchester, Manchester, UK; 32 Infectious Diseases Department, Ospedale Civile SS. Giovanni e Paolo, Venice, Italy; 33 Intensive Care Department, Vrije Universiteit Brussel (VUB), Universitair Ziekenhuis Brussel (UZ Brussel), Brussels, Belgium; 34 G. Gennimatas General Hospital of Thessaloniki, Thessaloniki, Greece; 35 Université Paris Diderot/Hopital Bichat-Réanimation Medicale et Des Maladies Infectieuses, Paris, France; 36 UMR 1137-IAME Team 5-DeSCID: Decision SCiences in Infectious Diseases, Control and Care, Inserm/Univ Paris Diderot, Sorbonne Paris Cité, Paris, France; 37 Institute of Infectious Diseases, Fondazione Policlinico Universitario A. Gemelli IRCCS – Università Cattolica del Sacro Cuore, Rome, Italy; 38 Department of Intensive Care, University Medical Center Groningen, Groningen, the Netherlands; 39 Pole Anesthésie-Réanimation-SAMU, Rouen University Hospital, Rouen, France; 40 Department of Public Health and Infectious Diseases, Sapienza University of Rome, Rome, Italy; 41 Sorbonne Université, Assistance Publique-Hôpitaux de Paris, Service de réanimation médico-chirurgicale, Hôpital Tenon, 75020 Paris, France; 42 Department of General Internal Medicine, Medical Intensive Care Unit, University Hospitals Leuven, Leuven, Belgium; 43 Université de Paris, INSERM UMR 1152, Paris, France

**Keywords:** ICU, Candidemia, Candidiasis, *Candida*, Abdominal candidiasis, Incidence

## Abstract

**Background:**

The objective of this study was to assess the cumulative incidence of invasive candidiasis (IC) in intensive care units (ICUs) in Europe.

**Methods:**

A multinational, multicenter, retrospective study was conducted in 23 ICUs in 9 European countries, representing the first phase of the candidemia/intra-abdominal candidiasis in European ICU project (EUCANDICU).

**Results:**

During the study period, 570 episodes of ICU-acquired IC were observed, with a cumulative incidence of 7.07 episodes per 1000 ICU admissions, with important between-center variability. Separated, non-mutually exclusive cumulative incidences of candidemia and IAC were 5.52 and 1.84 episodes per 1000 ICU admissions, respectively. Crude 30-day mortality was 42%. Age (odds ratio [OR] 1.04 per year, 95% CI 1.02–1.06, *p* < 0.001), severe hepatic failure (OR 3.25, 95% 1.31–8.08, *p* 0.011), SOFA score at the onset of IC (OR 1.11 per point, 95% CI 1.04–1.17, *p* 0.001), and septic shock (OR 2.12, 95% CI 1.24–3.63, *p* 0.006) were associated with increased 30-day mortality in a secondary, exploratory analysis.

**Conclusions:**

The cumulative incidence of IC in 23 European ICUs was 7.07 episodes per 1000 ICU admissions. Future in-depth analyses will allow explaining part of the observed between-center variability, with the ultimate aim of helping to improve local infection control and antifungal stewardship projects and interventions.

## Background

Invasive candidiasis (IC) can develop in adult patients admitted in intensive care units (ICUs), with a significant impact on morbidity, mortality, and healthcare costs [1–4]. The most frequent clinical forms of IC in critically ill patients are candidemia and intra-abdominal candidiasis (IAC), which affect up to 5% of all ICU admissions [2, 5].

In the last 10 years, other series addressed specifically the cumulative incidence of IC in the ICU setting [6–10]. The majority of those studies was limited to candidemia, some were limited to one or a few countries, and some occasionally included cases not representing true infections but colonization [6–10].

The aim of the present multinational study was to expand our knowledge of the cumulative incidence of ICU-acquired IC in Europe.

## Material and methods

The present multicenter, retrospective study was conducted from January 2015 to December 2016 in 23 ICUs in 22 large tertiary care European hospitals (9 in Italy, 4 in France, 2 in Greece, 1 in Belgium, 1 in Czech Republic, 1 in Germany, 1 in Ireland, 1 in Portugal, 1 in Spain, 1 in The Netherlands, and 1 in the UK). All patients who developed an episode of candidemia or a microbiologically documented IAC [11] during their stay in the ICU (at least 48 h after admission) were included in the study.

The primary objective of the study was to assess the cumulative incidence of ICU-acquired IC in European ICUs. Secondary objectives were (i) to assess the independent impact of center-level factors on the cumulative incidence of ICU-acquired IC and (ii) to assess factors associated with crude 30-day mortality in patients with ICU-acquired IC.

The entire EUCANDICU project consists of two phases: (i) a first study describing the cumulative incidence of ICU-acquired IC in Europe, reported in the present paper; (ii) a second, case-control study to assess patient-level predictors of ICU-acquired IC, which will be conducted and published subsequently.

### Definitions

ICU-acquired IC was defined as candidemia or IAC with signs and symptoms of infection developing at least 48 h after ICU admission. Candidemia and IAC were defined according to previously published definitions [6, 11]. More in detail, candidemia was defined as the presence of at least one positive blood culture for *Candida* spp. in patients with signs and symptoms of infection. IAC was defined as the presence of at least one of the following: (i) *Candida* detection by direct microscopy or growth in culture from necrotic or purulent intra-abdominal specimens obtained by percutaneous aspiration or during surgery; (ii) growth of *Candida* from the bile or intra-biliary duct devices, plus biopsy of intra-abdominal organs; (iii) growth of *Candida* from blood cultures in the presence of secondary or tertiary peritonitis in the presence of no other pathogens; (iv) growth of *Candida* from drainage tubes inserted less than 24 h before culture sampling [11]. In case of multiple episodes of IC in the same patient, a subsequent event was considered as independent if developing at least 30 days after the last positive culture related to the previous episode. Crude 30-day mortality was defined as death within 30 days from the onset of signs and symptoms of ICU-acquired IC. All patients suitable for inclusion were identified starting from the laboratory databases of the participating hospitals, and subsequent review of clinical records.

### Statistical analysis

The cumulative incidence of ICU-acquired IC was measured as the number of episodes per 1000 ICU admissions. The impact of center-level factors on the cumulative incidence of ICU-acquired IC was assessed by means of a multivariable generalized Poisson mixed model with center as a random intercept, after having verified the absence of overdispersion in count data. Subgroup analyses were also conducted according to the type of ICU-acquired IC (candidemia and IAC).

Demographic and clinical characteristics of patients are presented with number and percentage for categorical variables and median and interquartile range (IQR) for continuous variables. Their possible association with crude 30-day mortality was firstly tested through univariable logistic regression. Then, factors potentially associated with the outcome in univariable comparisons (*p* < 0.10) were included in an initial multivariable regression model and further selected for the final multivariable model (model A) by means of a stepwise backward procedure based on the Akaike information criterion. Only the first IC episode per patient was considered for this analysis. Variables included in model A were also included in an additional multivariable mixed logistic regression model (model B), with center as a random intercept.

All the analyses were conducted using R Statistical Software 3.5.2 (R Foundation for Statistical Computing, Vienna, Austria). Mixed models were built using the glmer function in the lme4 package.

## Results

### Primary analysis—cumulative incidence

**Fig. 1:**
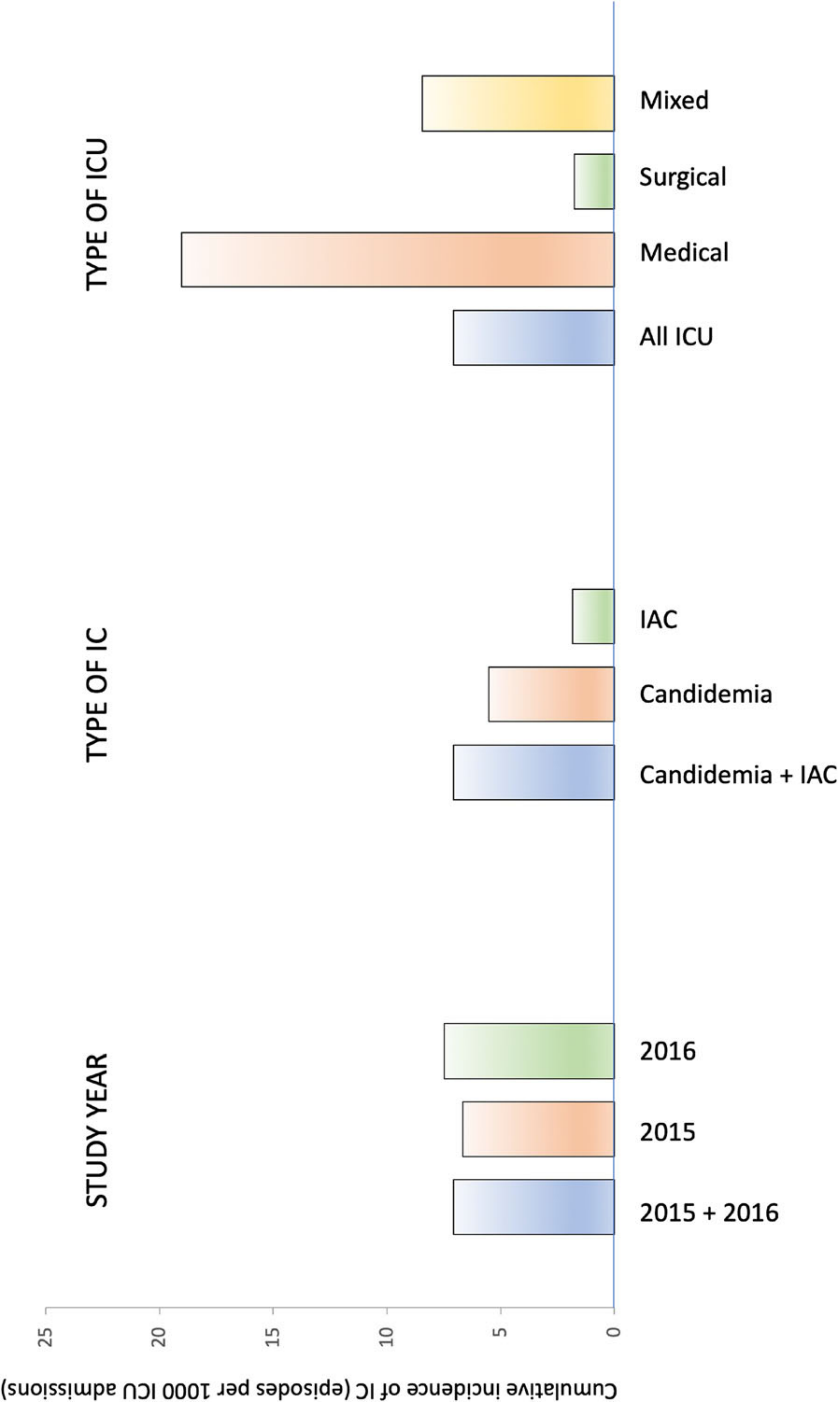
Cumulative incidence of ICU-acquired invasive candidiasis. IAC, intra-abdominal candidiasis; IC, invasive candidiasis; ICU, intensive care unit

During the study period, the 23 ICUs (median number of beds 18, interquartile range 14–43) had 80,645 admissions and 570 episodes of ICU-acquired IC, corresponding to an incidence of 7.07 episodes per 1000 ICU admissions (6.67 and 7.47 in 2015 and 2016, respectively). Separated, non-mutually exclusive incidences of candidemia and IAC were 5.52 and 1.84 episodes per 1000 ICU admissions, respectively. As shown in Table 1, in subgroup comparisons, admission to a surgical ICU was associated with lower incidence when compared with a medical ICU (cumulative incidence ratio 0.10, 95% CI 0.01–0.76 for surgical vs. medical, *p* 0.022). The observed random effects variance testified to the presence of between-center variability in the cumulative incidence of IC that was unexplained by the explored, fixed center-level predictors (Table 1 legend). A graphic representation of the cumulative incidence of IC is also available in Fig. 1.

**Table 1:** Multivariable analysis of center-level factors potentially associated with changes in the cumulative incidence of invasive candidiasis in European ICUs

Center-level variables*	Number of IC episodes	Number of ICU admissions	Cumulative incidence (IC episodes/1000 ICU admissions)	CIR (95% CI)	*p*
Year of study					0.313
2015	271	40,642	6.67	Ref	
2016	299	40,003	7.47	1.18 (0.85–1.63)	
Type of ICU					0.073
Medical (*n* = 5)	149	7828	19.03	Ref	
Surgical (*n* = 3)	51	29,087	1.75	0.10 (0.01–0.76)^§^	
Mixed (medical plus surgical, *n* = 15)	370	43,730	8.46	0.40 (0.10–1.63)	

The sample size was of 46 observations (2 for each of the 23 participating ICUs, one in 2015 and one in 2016). Non-independence was accounted by adding center as random effect. The model also included an interaction term (year of study × type of ICU), with *p* for interaction 0.761. Results of the main model (including both candidemia and IAC) were confirmed in a subgroup analysis including only patients with candidemia and not IAC (*n* = 422), suggesting that the observed increased cumulative incidence of IC in medical ICU vs. surgical ICU was mainly due to candidemia and not IAC: year of study (CIR 1.09, 95% CI 0.77–1.55, *p* 0.619), type of ward (*p* 0.005, with CIR 0.03 for surgical vs. medical, 95% CI 0.00–0.31, and CIR 0.34 for mixed vs. medical, 95% CI 0.07–1.55), year of study × type of ward (*p* for interaction 0.782), center as random intercept (standard deviation of the random effect = 1.410; model *β*_0_ = − 3.937). Results of the subgroup analysis of patients with IAC (*n* = 148) were as follows: year of study (CIR 1.77, 95% CI 0.79–4.21, *p* 0.177), type of ward (*p* 0.798, with CIR 0.94 for surgical vs. medical, 95% CI 0.07–12.51, and CIR 0.82 for mixed vs. medical, 95% CI 0.12–5.37), year of study × type of ward (*p* for interaction 0.290), center as random intercept (standard deviation of the random effect = 1.565; model *β*_0_ = − 6.285). Stratified cumulative incidences for countries in the entire study period was as follows: Italy (2 medical and 7 mixed ICUs), 89.62 episodes per 1000 ICU admissions (range 1.20–114.21); France (2 surgical, 1 medical, and 1 mixed ICUs), 11.85 episodes per 1000 ICU admissions (range 0.62–27.63); Greece (1 medical and 1 mixed ICUs), 30.79 per 1000 ICU admissions (range 7.50–45.73); Belgium (1 medical ICU), 9.28 episodes per 1000 ICU admissions; Czech Republic (1 surgical ICU), 0.90 per 1000 ICU admissions; Germany (1 mixed ICU), 42.43 episodes per 1000 hospital admissions; Ireland (1 mixed ICU), 5.63 episodes per 1000 ICU admissions; Portugal (1 mixed ICU), 9.33 episodes per 1000 ICU admissions; Spain (1 mixed ICU), 10.46 episodes per 1000 ICU admissions; The Netherlands (1 mixed ICU), 2.29 episodes per 1000 ICU admissions; UK (1 mixed ICU), 41.67 episodes per 1000 ICU admissions

*CI* confidence intervals, *CIR* cumulative incidence ratio, *IC* invasive candidiasis, *ICU* intensive care unit, *IQR* interquartile range

*The model also includes center as a random intercept (standard deviation of the random effect = 1.293; model *β*_0_ = − 3.763)

^§^*p* = 0.022 for the subgroup comparison surgical vs. medical

### Secondary analysis—predictors of mortality

An additional, secondary analysis of predictors of mortality was conducted in the subgroup of patients for whom patient-level clinical data was registered (mostly cases of IC meeting the following inclusion criterion for the future case-control study: availability of controls with the same length of ICU stay without IC). In this regard, clinical data were available for 330/570 cases of IC (58%). The median age of patients was 66 (IQR 55–75), and 60% were males (198/330). Candidemia, IAC, and IAC plus candidemia accounted for 65% (215/330), 29% (97/330), and 5% (18/330) of episodes, respectively. *C. albicans* was the most frequently isolated species (189/330, 57%), followed by *C. glabrata* (68/330, 21%) and *C. parapsilosis* (42/330, 13%). Crude 30-day mortality was 42% (137/330). Table 2 shows univariable and multivariable analyses of predictors of crude 30-day mortality. In the final multivariable model (model A), age (odds ratio [OR] 1.04 per year, 95% CI 1.02–1.06, *p* < 0.001), severe hepatic failure (OR 3.25, 95% 1.31–8.08, *p* 0.011), SOFA score at the onset of IC (OR 1.11 per point, 95% CI 1.04–1.17, *p* 0.001), and septic shock (OR 2.12, 95% CI 1.24–3.36, *p* 0.006) were associated with increased 30-day mortality. As reported in the legend of Table 2, the additional multivariable model with center as random intercept (model B) confirmed the results obtained in model A.

**Table 2:** Univariable and multivariable analyses of factors associated with crude 30-day mortality in patients with ICU-acquired IC

Variable	Total of patients (%) *n* = 330 (100)	Non-survivors (%) *n* = 137 (42)	Survivors (%) *N* = 193 (58)	Univariable analysis		Multivariable analysis*	
				OR (95% CI)	*p*	OR (95% CI)	*p*
Demographics							
Age in years, median (IQR)	66 (55–75)	68 (59–77)	64 (51–73)	1.03 (1.01–1.05)	0.001	1.04 (1.02–1.06)	< 0.001
Male gender	198 (60)	84 (61)	114 (59)	1.10 (0.70–1.72)	0.681		
Medical history							
Diabetes mellitus	73 (22)	34 (25)	39 (20)	1.30 (0.77–2.20)	0.321		
COPD	44 (13)	22 (16)	22 (11)	1.49 (0.79–2.81)	0.222		
End-stage chronic renal disease	59 (18)	35 (26)	24 (12)	2.42 (1.36–4.29)	0.003	1.82 (0.96–3.45)	0.068
Severe hepatic failure^a^	29 (9)	18 (13)	11 (6)	2.50 (1.14–5.49)	0.022	3.25 (1.31–8.08)	0.011
Solid tumor	94 (28)	40 (29)	54 (28)	1.06 (0.65–1.72)	0.809		
Hematological malignancy	16 (5)	5 (4)	11 (6)	0.63 (0.21–1.85)	0.397		
Solid organ transplant	18 (5)	5 (4)	13 (7)	0.52 (0.18–1.51)	0.231		
Steroid treatment	43 (13)	20 (15)	23 (12)	1.26 (0.66–2.41)	0.477		
Immunosuppressants other than steroids	30 (9)	13 (9)	17 (9)	1.09 (0.51–2.32)	0.832		
Age-adjusted Charlson score	6 (3–7)	6 (5–8)	5 (3–7)	1.16 (1.07–1.25)	< 0.001		
Recent exposures (within 30 days)							
Previous abdominal surgery	174 (53)	65 (47)	109 (56)	0.70 (0.45–1.08)	0.106		
Previous antibacterial therapy	226 (68)	102 (74)	124 (64)	1.62 (1.00–2.63)	0.050	1.53 (0.89–2.64)	0.124
Previous echinocandins	35 (11)	12 (9)	23 (12)	0.71 (0.34–1.48)	0.360		
Previous azoles	53 (16)	22 (16)	31 (16)	1.00 (0.55–1.82)	0.999		
Previous amphotericin B	5 (2)	4 (3)	1 (1)	5.77 (0.64–52.24)	0.119		
Baseline variables**							
SOFA score, median (IQR)	9 (5–12)	10 (7–13)	7 (4–10)	1.16 (1.10–1.22)	< 0.001	1.11 (1.04–1.17)	0.001
SAPS II score, median (IQR)	48 (35–64)	55 (40–72)	43 (31–56)	1.03 (1.02–1.04)	< 0.001		
Length of ICU stay in days (IQR)	8 (3–19)	9 (3–20)	8 (3–18)	1.00 (0.99–1.01)	0.469		
WBC (cells × 10^9^/L), median (IQR)	13.6 (8.2–20.2)	13.2 (7.5–20.0)	13.9 (8.8–20.7)	0.99 (0.98–1.01)	0.497		
AKI^§^	157 (48)	81 (59)	76 (39)	2.23 (1.43–3.48)	< 0.001		
Infection variables							
Type of IC					0.193		
IAC	97 (29)	34 (25)	63 (33)	(ref)			
Candidemia	215 (65)	97 (71)	118 (61)	1.52 (0.93–2.50)			
IAC plus candidemia	18 (5)	6 (4)	12 (6)	0.93 (0.32–2.69)			
*Candida* species					0.866		
*Candida albicans*	162 (49)	65 (47)	97 (50)	(ref)			
Non-*Candida albicans*^§§^	141 (43)	60 (40)	81 (42)	1.11 (0.70–1.75)			
*Candida albicans* plus non-*Candida albicans*^§§§^	27 (8)	12 (9)	15 (8)	1.19 (0.53–2.72)			
Presence of septic shock^b^	165 (50)	91 (66)	74 (38)	3.18 (2.01–5.03)	< 0.001	2.12 (1.24–3.63)	0.006
Presence of endocarditis	8 (2)	3 (2)	5 (3)	0.84 (0.20–3.58)	0.816		
Fluconazole resistance^c^	66 (24)	28 (25)	38 (23)	1.01 (0.65–1.99)	0.665		
Early treatment variables***							
Adequate source control within 24 h^d^	205 (62)	73 (53)	132 (68)	0.53 (0.34–0.83)	0.006	0.65 (0.39–1.07)	0.093
Adequate empiric antifungals within 24 h^e^	93 (36)	35 (36)	58 (36)	0.97 (0.57–1.63)	0.902		

Results are presented as *n* (%) unless otherwise indicated. *AKI* acute kidney injury, *COPD* chronic obstructive pulmonary disease, *CVC* central venous catheter, *IC* invasive candidiasis, *IAC* intra-abdominal candidiasis, *ICU* intensive care unit, *IQR* interquartile range, *SAPS* simplified acute physiology score, *SOFA* sequential organ failure assessment, *WBC* white blood cells

*Only results for variables retained in the final multivariable model (model A) are presented. Variables included in model A were also included in an additional generalized linear multivariable mixed logistic regression model with center as a random intercept (model B; standard deviation of the random effect = 0.311; model *β*_0_ = −4.329), the results of which were in line with those of model A: age (OR 1.04, 95% CI 1.02–1.06, *p* < 0.001); end-stage chronic renal disease (OR 1.83, 95% IC 0.95–3.52, *p* 0.070), severe liver failure (OR 3.41, 95% CI 1.33–8.73, *p* 0.010); previous antibacterial therapy (OR 1.51, 95% CI 0.86–2.63, *p* 0.148); SOFA score (OR 1.11, 95% CI 1.04–1.18, *p* 0.001); septic shock (OR 2.09, 95% CI 1.21–3.62, *p* 0.008), adequate source control within 24 h (OR 0.64, 95% CI 0.38–1.08, *p* 0.095). The Akaike information criterion (AIC) values for model A and model B were 391.1 and 392.5, respectively

**At the onset of signs and symptoms of IC

***The present exploratory model was not developed to comprehensively assess the overall impact of antifungal therapy and/or adequate source control (including those performed beyond 24 h after the onset of symptoms), which needs further dedicated investigation to be reliably evaluated

^§^ClCr < 60 mL/min

^§§^*C*. *glabrata* (*n* = 52), *C. parapsilosis* (*n* = 38), *C. tropicalis* (*n* = 18), *C. krusei* (n = 14), *C. dubliniensis* (*n* = 4), other species with lower frequency (*n* = 5), more than one non-*Candida albicans* spp. concomitantly (*n* = 10)

^§§§^*C. albicans* plus *C. glabrata* (*n* = 16), *C. albicans* plus *C. parapsilosis* (*n* = 4), other combinations with lower frequency (*n* = 7)

^a^Severe hepatic failure was defined as liver cirrhosis according to histology or in the presence of a clinical diagnosis supported by laboratory, endoscopy, and radiologic findings

^b^Septic shock was defined as hypotension not responding to fluid therapy and requiring vasoactive agents

^c^Information available (i.e., fluconazole tested) for 274/330 patients (83%)

^d^Source control was considered adequate in the following cases: (i) not necessary, (ii) devices or foreign body removal, (iii) drainage of infected fluid collections, (iv) debridement of infected solid tissue, and (v) definitive measures to correct anatomic derangements resulting in ongoing microbial contamination

^e^From the onset of signs and symptoms of IC. Empiric treatment was considered adequate when the infecting organism was ultimately shown to be susceptible to the empirically administered antifungal. This analysis was conducted in the subgroup of patients not receiving empirical antifungal or receiving empirical antifungals for treating *Candida* spp. for which susceptibility test results were subsequently available (257/330, 78%)

## Discussion

This is the largest multinational study assessing the cumulative incidence of ICU-acquired IC in Europe to date. Based on our report, the incidence of IC in European ICUs was 7.07 episodes per 1000 ICU admissions, with crude 30-day mortality of 42%.

Our results are similar to those of the large point-prevalence EPIC II study, which previously reported a prevalence of 6.87 episodes of candidemia per 1000 ICU patients, although we recorded a slightly lower cumulative incidence of candidemia (5.52 episodes per 1000 ICU admissions) [6]. In addition, our analysis indicated two center-level factors that may influence local incidences: (i) the type of ICU, with medical ICUs being associated with increased incidence vs. surgical ICUs in subgroup comparisons, and (ii) the presence of random between-center variability, although likely relying in part on unexplored but exhaustive center-level factors. It is also worth noting that some unexplored center-level factors (e.g., cumulative days at risk, antifungal prophylaxis policies), together with the low number (and thus low generalizability) of ICU wards included in the subgroup comparison surgical vs. medical (3 vs. 5, respectively), might have influenced the unexpected but significant observation of a lower cumulative incidence of IC in surgical ICUs, which calls for future confirmatory studies specifically addressing this aspect.

In a secondary analysis, we confirmed the importance of baseline factors (age, severe hepatic failure) and severity of disease/clinical presentation (SOFA score, septic shock) in unfavorably influencing the prognosis of ICU-acquired IC [2, 12]. However, we could not explore the impact on mortality of both source control and antifungal therapy performed/administered beyond 24 h after the onset of IC. Dedicated studies and models aimed at primarily evaluating these variables are necessary to comprehensively assess their effect.

An important limitation of our study is the limited number of assessed, potential center-level predictors of cumulative incidence. Further studies with a higher number of participating ICUs are necessary for allowing inclusion of more variables in the model. Other main limitations of our study are the lack of a control group (patients without IC) for assessing patient-level predictors of IC and the inherent possibility of having considered some contamination as IAC. As regards the former, it should be noted that the main goal in which we were interested in this first phase was to provide a center-level perspective on the cumulative incidence of ICU-acquired IC, by describing its burden and assessing the possible effect of fixed and random center-level predictors. Although it will ultimately add to the discussion, the assessment of patient-level predictors is an independent and different analysis, which will be the core of the future case-control study. As regards the possibility of including contaminations, it is worth noting that we used a consensus definition for minimizing the number of contaminations misidentified as IAC [11]. Another limitation is that most data came from a few countries (e.g., 9/23 participating ICUs are in Italy), thus preventing us from reliably assessing the existence of any possible between-country variability impacting on the cumulative incidence of ICU-acquired IC. Finally, it should be acknowledged that the impact on mortality of some covariates (e.g., COPD) may depend on their degree of severity, which was not registered.

## Conclusions

Global incidence of IC in 23 European ICUs was 7.07 episodes per 1000 ICU admissions. Future in-depth analyses will allow explaining part of the observed between-center variability, with the ultimate aim of helping to improve local infection control and antifungal stewardship interventions.

## Data Availability

The datasets used and/or analyzed during the current study are available from the corresponding author on reasonable request.

## Abbreviations

CI: Confidence intervals
COPD: Chronic obstructive pulmonary disease
IAC: Intra-abdominal candidiasis
IC: Invasive candidiasis
ICU: Intensive care unit
OR: Odds ratio
SOFA: Sequential organ failure assessment

## References

[1] Bassetti M, Righi E, Ansaldi F, Merelli M, Scarparo C, Antonelli M (2015). A multicenter multinational study of abdominal candidiasis: epidemiology, outcomes and predictors of mortality. Intensive Care Med.

[2] Bassetti M, Righi E, Ansaldi F, Merelli M, Trucchi C, De Pascale G (2014). A multicenter study of septic shock due to candidemia: outcomes and predictors of mortality. Intensive Care Med.

[3] Bouza E, Munoz P (2008). Epidemiology of candidemia in intensive care units. Int J Antimicrob Agents.

[4] Leroy O, Bailly S, Gangneux JP, Mira JP, Devos P, Dupont H (2016). Systemic antifungal therapy for proven or suspected invasive candidiasis: the AmarCAND 2 study. Ann Intensive Care.

[5] Guery BP, Arendrup MC, Auzinger G, Azoulay E, Borges Sá M, Johnson EM (2009). Management of invasive candidiasis and candidaemia in adult non-neutropenic intensive care unit patients. Part I. epidemiology and diagnosis. Intensive Care Med.

[6] Kett DH, Azoulay E, Echeverria PM, Vincent JL (2011). Extended Prevalence of Infection in ICU Study (EPIC II) Group of Investigators. Candida bloodstream infections in intensive care units: analysis of the extended prevalence of infection in intensive care unit study. Crit Care Med.

[7] Baldesi O, Bailly S, Ruckly S, Lepape A, L'Heriteau F, Aupee M (2017). ICU-acquired candidaemia in France: epidemiology and temporal trends, 2004-2013 - a study from the REA-RAISIN network. J Inf Secur.

[8] Vincent JL, Rello J, Marshall J, Silva E, Anzueto A, Martin CD (2009). International study of the prevalence and outcomes of infection in intensive care units. JAMA..

[9] Tortorano AM, Dho G, Prigitano A, Breda G, Grancini A, Emmi V (2012). Invasive fungal infections in the intensive care unit: a multicentre, prospective, observational study in Italy (2006-2008). Mycoses..

[10] Paiva JA, Pereira JM, Tabah A, Mikstacki A, de Carvalho FB, Koulenti D (2016). Characteristics and risk factors for 28-day mortality of hospital acquired fungemias in ICUs: data from the EUROBACT study. Crit Care.

[11] Bassetti M, Marchetti M, Chakrabarti A, Colizza S, Garnacho-Montero J, Kett DH (2013). A research agenda on the management of intra-abdominal candidiasis: results from a consensus of multinational experts. Intensive Care Med.

[12] Bassetti M, Peghin M, Carnelutti A, Righi E, Merelli M, Ansaldi F (2017). Clinical characteristics and predictors of mortality in cirrhotic patients with candidemia and intra-abdominal candidiasis: a multicenter study. Intensive Care Med.

